# Upregulation of Mcl-1S Causes Cell-Cycle Perturbations and DNA Damage Accumulation

**DOI:** 10.3389/fcell.2020.543066

**Published:** 2020-09-25

**Authors:** Alena Y. Streletskaia, Viacheslav V. Senichkin, Tatiana A. Prikazchikova, Timofei S. Zatsepin, Boris Zhivotovsky, Gelina S. Kopeina

**Affiliations:** ^1^Faculty of Medicine, MV Lomonosov Moscow State University, Moscow, Russia; ^2^Skolkovo Institute of Science and Technology, Skolkovo, Russia; ^3^Institute of Environmental Medicine, Karolinska Institutet, Stockholm, Sweden

**Keywords:** apoptosis, cell cycle, splicing switch, Mcl-1, BH3-mimetics

## Abstract

As an important regulator of apoptosis, Mcl-1 protein, a member of the Bcl-2 family, represents an attractive target for cancer treatment. The recent development of novel small molecule compounds has allowed Mcl-1-inhibitory therapy to proceed to clinical trials in cancer treatment. However, the possible adverse effects of either direct inhibition of Mcl-1 or upregulation of Mcl-1S, proapoptotic isoform resulting from alternative splicing of Mcl-1, remain unclear. Here, we investigated changes in Mcl-1S levels during cell cycle and the cell cycle-related functions of Mcl-1 isoforms to address the above-mentioned concerns. It was shown that an anti-mitotic agent monastrol caused accumulation of Mcl-1S mRNA, although without increasing the protein level. In contrast, both mRNA and protein levels of Mcl-1S accrued during the premitotic stages of the normal cell cycle progression. Importantly, Mcl-1S was observed in the nuclear compartment and an overexpression of Mcl-1S, as well as knockdown of Mcl-1, accelerated the progression of cells into mitosis and resulted in DNA damage accumulation. Surprisingly, a small molecule inhibitor of Mcl-1, BH3-mimetic S63845, did not affect the cell cycle progression or the amount of DNA damage. In general, upregulated Mcl-1S protein or genetically inhibited Mcl-1L were associated with the cell cycle perturbations and DNA damage accumulation in normal and cancer cells. At the same time, BH3-mimetic to Mcl-1 did not affect the cell cycle progression, suggesting that direct inhibition of Mcl-1 is devoid of cell-cycle related undesired effects.

## Introduction

The Myeloid cell leukemia-1 (*MCL1*) gene was initially discovered in maturing human myeloid cells and revealed a significant sequence similarity to *BCL2* (Kozopas et al., 1993). As one of the *BCL2* family members, *MCL1* possesses the Bcl-2 homology (BH) regions, whose composition determines the antiapoptotic or proapoptotic properties of the corresponding proteins (Letai et al., 2002). *MCL1* is known to give rise to at least two alternative splicing (AS) isoforms – antiapoptotic Mcl-1L (traditionally abbreviated as Mcl-1) and proapoptotic Mcl-1S. Mcl-1L protein contains BH1-BH4 motifs, three of which form a BH3-binding groove for sequestration of proapoptotic proteins, whereas only BH3 and BH4 motifs are found in Mcl-1S, thus establishing its proapoptotic characteristics (Senichkin et al., 2019). Moreover, it was shown that Mcl-1L is the only Bcl-2 family protein that binds to Mcl-1S (Bae et al., 2000; Stewart et al., 2010).

Mcl-1S transcripts were first detected in the placenta and some human cell lines (Bae et al., 2000; Bingle et al., 2000). Due to AS, Mcl-1S mRNA lacks exon 2 of the *MCL1* gene. However, the presence of exon 1 causes retention of a large N-terminal region, which is identical to that of Mcl-1L (Senichkin et al., 2019). This N-terminus includes a mitochondrial targeting sequence (Perciavalle et al., 2012), putative nuclear localization signal (Ye et al., 2017) and PEST clusters (rich in proline, glutamic acid, serine, and threonine) (Kozopas et al., 1993). PEST motifs define a high turnover rate (e.g., the Mcl-1L half-life is estimated to be less than 1 h) (Rogers et al., 1986; Chao et al., 1998) and, therefore, enable the rapid regulation of protein levels in response to internal and external stimuli (Kozopas et al., 1993; Senichkin et al., 2020). Being timely regulated, Mcl-1L can not only perform functions that are related to apoptosis but also control non-apoptotic processes, such as the cell cycle (Senichkin et al., 2019).

Mcl-1L interacts with some regulatory proteins of the cell cycle and division (Fujise et al., 2000; Jamil et al., 2008; Wang et al., 2014). For instance, Mcl-1L is capable of binding to PCNA, a co-factor of DNA-Pol δ, thus impeding S-phase progression (Fujise et al., 2000). Mcl-1L also modulates phosphorylation of Chk1, both in response to genotoxic stress and upon normal DNA replication, and favors arrest in G2/M phase for DNA repair (Jamil et al., 2008, 2010; Pawlikowska et al., 2010; Mattoo et al., 2017). Cells deficient in Mcl-1L manifest cell cycle aberrations and DNA damage accumulation (Pawlikowska et al., 2010; Mattoo et al., 2017). Conversely, Mcl-1L overexpression decelerates mitotic progression and cell proliferation rates (Zinkel et al., 2006; Sloss et al., 2016). Moreover, Mcl-1 pre-mRNA AS was found to be controlled by proteins related to the cell cycle process (mainly associated with G2/M-transition and spindle formation) (Moore et al., 2010). In particular, siRNA-mediated knockdown of the factors CHTOG, TPX2, NDC80, CDK1, AURKA, PLK1, CDC5L, and others induced switching of Mcl-1 pre-mRNA splicing toward the Mcl-1S isoform (Moore et al., 2010).

However, currently, there is no data on the cell cycle functions of Mcl-1S. Furthermore, potential implications of Mcl-1 inhibition for the cell cycle in tumor and normal cells remain poorly investigated. These issues are important in the context of adverse effects of Mcl-1 targeted therapy and also require prompt examination, considering the recent advances in the development of novel drugs that antagonize Mcl-1 (so-called “BH3-mimetics” that imitate the action of proapoptotic BH3-domains) (Kotschy et al., 2016; Caenepeel et al., 2018; Tron et al., 2018) and the progression of AS modulators into clinical trials (Seiler et al., 2018).

## Materials and Methods

### Cell Culture and Treatment

The human embryonic kidney HEK293T cells, fetal lung fibroblasts IMR90, cervical adenocarcinoma HeLa, ovarian carcinoma Caov-4, colorectal carcinoma HCT116, and non-small cell lung carcinoma U1810 cell lines were cultured in DMEM or RPMI-1640 medium (Gibco, Paisley, United Kingdom), supplemented with 10% FBS (Gibco), 1% sodium pyruvate (PanEco, Moscow, Russia) and Antibiotic-Antimycotic (Gibco) in a humidified atmosphere of 5% CO_2_ at 37°C. The cultures were re-plated every 3–4 days to maintain an asynchronous population and optimal growth. For DNA damage induction, HEK293T cells were treated with cisplatin, doxorubicin, or etoposide (all from Teva, Yaroslavl, Russia) for the indicated concentrations and periods. The spliceosome inhibitor FR901464 (kindly provided by Dr. Kazuo Shin-ya, National Institute of Advanced Industrial Science and Technology, Tokyo, Japan) was used in experiments as a positive control for Mcl-1 pre-mRNA switching toward the Mcl-1S isoform at a concentration of 10 nM, if not stated differently (Supplementary Figure 1B) (Kaida et al., 2007; Laetsch et al., 2014; Larrayoz et al., 2016). For the cell cycle delay, HEK293T cells were exposed to a mitotic inhibitor Monastrol (Abcam, Cambridge, MA, United States) at different concentrations, as indicated in the figures. The selective inhibition of Mcl-1L was achieved using a BH3-mimetic S63845 (Active Biochem, Hong Kong, China).

### Cell Cycle Synchronization

Growing cells were synchronized at the G1/S boundary using a double thymidine block. Cells at 40–50% confluence were treated with 1.5–2.5 mM thymidine (Sigma-Aldrich, St. Louis, MO, United States) for 18 h, then washed twice with PBS (PanEco), incubated in the regular medium for 9 h, and subjected to a second incubation with thymidine for 18 h. Samples were collected immediately after the synchronization (0 h) or at the indicated periods after the cell cycle release, implemented by incubation of cells in a fresh growth medium.

### Lipofectamine-Mediated Transfection

Plasmid pcDNA3-hMcl-1S was generously provided by Dr. Elizabeth Henson (the Gibson laboratory, University of Manitoba, Winnipeg, MB, Canada). siRNA against MCL1 transcripts (the sense and antisense sequences: 5′-GCATCGAACCATTAGCAGAdTdT-3′ and 5′-TCTGCTAATG GTTCGATGCdTdT-3′, respectively) were synthesized by us. For this reason, chemically modified siRNA targeting human Mcl-1 mRNA (NCBI GenBank accession codes NM_001197320.1, NM_182763.2, NM_021960.5) with the lowest off-target potential, including a minimal overlap with miRNA seed regions to avoid miRNA-like activity and decreased capacity to activate innate immunity, were designed (Amarzguioui and Prydz, 2004; Reynolds et al., 2004; Ui-Tei et al., 2008). Initially, the generated siRNA was ranked based on the number of mismatches in the seed region, mismatches in the non-seed region, and mismatches in the cleavage site position. The five best scored siRNAs were synthesized using the phosphoramidite approach, purified by IE-HPLC, and verified by LC-MS followed by strand annealing as previously described (Noll et al., 2011). Pyrimidine nucleotides upstream to adenosine residues were replaced with 2′-*O*-methyl analogs and a phosphorothioate linkage was introduced between two nucleotides at the 3′-end in both siRNA strands to improve nuclease stability. The control siRNA targeted the Firefly Luciferase gene. siRNAs were tested *in vitro* and then two duplexes with the highest efficacy were chosen for further work. The plasmid (final concentration: 1 μg/mL) or siRNA (50 nM) transfections were performed using Lipofectamine LTX Reagent with PLUS Reagent (Invitrogen, Carlsbad, CA, United States) or Lipofectamine RNAiMAX Transfection Reagent (Invitrogen), respectively, following the manufacturer’s guidelines.

### Western Blot Analysis and Antibodies

Protein concentrations were determined using the Pierce BCA Protein Assay Kit (Thermo Scientific, Rockford, IL, United States) as described by the manufacturer. Samples were mixed with Laemmli loading buffer (250 mM Tris-HCl, pH 6.8, 5% SDS, 50% glycerol, 25% β-mercaptoethanol, 0.1% bromophenol blue), boiled for 5 min and subjected to SDS-PAGE on a 12% resolving gel, followed by transfer to nitrocellulose membranes for 2 h at 110 V using Mini Trans-Blot cells (Bio-Rad, Hercules, CA, United States). After 1 h of blocking with 5% non-fat milk in TBS at room temperature and four times washing for 5 min each with TBST (0.05% Tween-20 in TBS), membranes were incubated overnight at 4°C with specific primary antibodies, and diluted to working concentrations according to the manufacturer’s guidelines. The membranes were then rinsed in TBST and probed with horseradish peroxidase-conjugated secondary antibodies (1:5000) for 1 h at room temperature. Chemiluminescence was produced using the Pierce ECL Western Blotting Substrate or SuperSignal West Dura Extended Duration Substrate (Thermo Scientific) and documented via the Chemi-Doc MP System (Bio-Rad). The Image Lab software (Bio-Rad) was used for densitometric analysis.

Primary antibodies to Chk1 (Cat #2360), cleaved PARP (9541), Cyclin B1 (4135), Na,K-ATPase (3010), Mcl-1 (5453), Phospho-cdc2 Tyr15 (9111), Phospho-Chk1 Ser345 (2341), and Phospho-Histone H2A.X Ser139 (2577) were purchased from Cell Signaling Technology (Beverly, MA, United States). Antibodies against Cdc2 (sc-54), Lamin B1 (sc-374015) were sourced from Santa Cruz Biotechnology (Santa Cruz, CA, United States). Anti-Erp29 antibodies were kindly provided by Dr. Souren Mkrtchian (Karolinska Institutet, Stockholm, Sweden). Anti-alpha tubulin (ab7291), PARP1 (ab137653), and vinculin (ab129002) primary antibodies, as well as goat anti-rabbit (ab97200) and anti-mouse (ab97265) secondary antibodies were obtained from Abcam (Cambridge, MA, United States).

### Quantitative Reverse Transcription PCR

Total RNA was obtained with the Extract RNA Reagent (Evrogen, Moscow, Russia), and reverse transcription was carried out using the MMLV RT kit (Evrogen) in accordance with the manufacturer’s instructions. mRNA expression (cDNA content) was measured by the Real-time CFX96 C1000 Touch system (Bio-Rad, Hercules, CA, United States) using the qPCRmix-HS SYBR (Evrogen) and specific primers (Table 1). The expression of Mcl-1S and Mcl-1L transcripts was normalized to *ACTB* or/and *RPL13A* as housekeeping genes and calculated using the 2^–ΔΔ*Ct*^ method.

**TABLE 1:** RT-qPCR primer sequences.

**Target transcript**	**Primer type**	**Primer sequence**
MCL-1L	Forward	5′-CAAAGCCAATGGGCAGGTCT-3′
	Reverse	5′-TTACGCCGTCGCTGAAAACA-3′
MCL-1S	Forward	5′-CAAAGCCAATGGGCAGGTCT-3′
	Reverse	5′-CTCCACAAACCCATCCTTGGAA-3′
RPL13A	Forward	5′-CTCAAGGTCGTGCGTCTGAA-3′
	Reverse	5′-ACGTTCTTCTCGGCCTGTTT-3′
ACTB	Forward	5′-AGAGCTACGAGCTGCCTGAC-3′
	Reverse	5′-AGCACTGTGTTGGCGTACAG-3′

*MCL-1L, myeloid cell leukemia-1 long isoform; MCL-1S, myeloid cell leukemia-1 short isoform; RPL13A, ribosomal protein L13a; ACTB, actin beta; RT-qPCR, quantitative reverse transcription PCR.*

The specificity of the amplification reaction was confirmed via amplicon sequencing (Supplementary Figure 1A) and qualitative PCR on 2% agarose gels (Supplementary Figure 1B). The spliceosome inhibitor FR901464 at a concentration of 10 nM (if not stated differently) was used as a positive control in the tests for the Mcl-1 pre-mRNA switching toward the Mcl-1S isoform (Supplementary Figures 1A–C).

### Cell Cycle Analysis by DNA Content

Cells were collected, rinsed twice with PBS, and resuspended in ice-cold 70% ethanol solution, which was added dropwise with gentle vortexing. The samples were kept overnight at −20°C, centrifuged at 800 *g* for 10 min and incubated with 100 μg/mL RNAse A (Invitrogen, Carlsbad, CA, United States) and 50 μg/mL propidium iodide (BD Biosciences, Franklin Lakes, NJ, United States) solution in PBS for 15 min at 37°C. The samples were run through the flow cytometer FACS Canto II (Becton Dickinson, Franklin Lakes, NJ, United States) and analyzed using Flow Jo software (Becton Dickinson).

For Figure 3, the following parameters were derived: the average rate of exit from G1-phase, the average rate of entry into G2/M phases, and the relation of these two values (exit/entry coefficient for S-phase progression). The average rate of exit from G1-phase was calculated as the absolute difference between the percentage of cells in G1 phase of the stated time point and the relative 0-h control (|dG1|), divided by time (dt). The average rate of entry into G2/M phase was counted as the difference between the percentage of cells in G2/M phases of the stated time point and the relative 0-h control (dG2/M), divided by time (dt). The exit/entry relation for S-phase was derived as the quotient of the average rate of entry into G2/M phases and the average rate of exit from G1 phase; the relation is expressed as the Mean ± SD for three independent experiments (Figure 3). The exit/entry coefficient for S phase reflects the rapidity of cells progressing through S phase. The chosen method of S phase calculations allowed integrating the varied exit from G1 phase and initial (0 h) difference in the G2/M portion. However, the method is based on the assumption that initial portion of the S-phase population is similar in treated cells and relative controls.

### Subcellular Fractionation

Nuclear and cytoplasmic fractionation was performed according to an approach previously described by us, which is based on cell lysis with NP-40 and washing the nuclei with a solution containing NP-40 (Prokhorova et al., 2018).

### Fluorescence Imaging

Cells were grown on 13-mm round glass coverlids (Thermo Scientific, Rockford, IL, United States) to 50% confluence and subjected to the double thymidine block as described above (see section “Cell Cycle Synchronization”). After synchronization (G1/S-phase) or a 6 h release from the block (G2/M-phase), the cells were fixed for 15 min in 4% paraformaldehyde solution (pH 7.4), washed four times with PBS and permeabilized with 0.1% Triton X-100 in PBS for 10 min. After rinsing in PBS, the specimens were incubated in blocking solution containing 2% BSA in PBST for 1 h at room temperature. Thereafter, primary antibodies against Mcl-1 (94296, Cell Signaling Technology, Beverly, MA, United States) diluted to 1:1000 in the blocking solution were incubated overnight at + 4°C. Cells were rinsed in PBST and immunostained with secondary antibodies Alexa Fluor 488 Conjugate (4412, Cell Signaling Technology) in the blocking solution at a dilution of 1:600 for 2 h at room temperature in the dark. After PBST washing, coverlids were incubated with 1 μg/mL DAPI (Invitrogen, Carlsbad, CA, United States) for 10 min, washed again in PBST and mounted. The samples were examined by confocal laser scanning microscopy (LSM 780, Zeiss, Jena, Germany) and analyzed using ImageJ software (NIH, Bethesda, MD, United States). For the colocalization analysis, at least three quadrants for each coverslip (about 100 cells) were analyzed using the ImageJ Colocalization Finder (NIH).

### Statistical Analysis

All the data were assessed for normality and homogeneity variance with the Shapiro–Wilk and Levene’s tests, respectively. Comparisons between experimental and control samples were obtained using the Student’s *t*-test for normally distributed results or Mann–Whitney’s *U*-test for the non-parametric data. Statistical difference was set at *p* < 0.05. The analysis was conducted using SPSS 23.0 (SPSS Inc., Chicago, IL, United States); the RT-qPCR data were processed with Bio-Rad CFX Software (Bio-Rad, Hercules, CA, United States).

## Results

### Mcl-1S Levels Increased During the G2/M Phases

Previous studies postulated a connection between the AS of Mcl-1 and cell cycle pathways. Thus, knockdown of some G2/M transition and mitotic regulators resulted in increased production of the Mcl-1S isoform (Moore et al., 2010). We hypothesized that arrest at G2/M or spindle-assembly checkpoints could be the primary triggers of AS favoring Mcl-1S synthesis. Since Mcl-1S is a proapoptotic protein (Bae et al., 2000; Stewart et al., 2010), such an AS switch could determine the apoptotic cell fate of the arrested (e.g., due to DNA damage or spindle malformation) cells.

**FIGURE 1:**
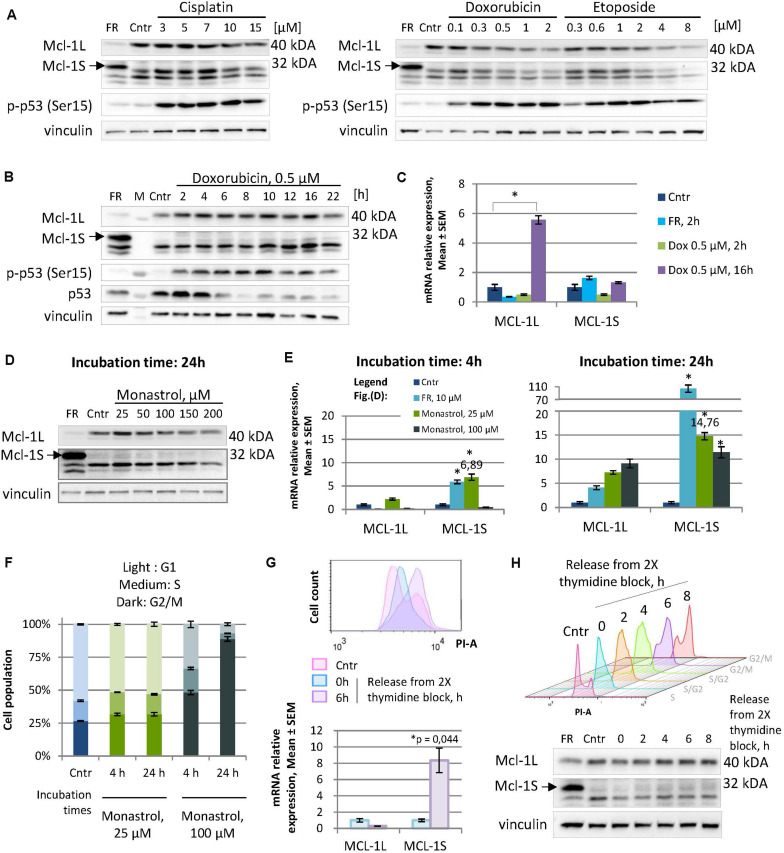
Not genotoxic stress but mild cell cycle perturbations cause an elevation in Mcl-1S mRNA and protein expression, revealing the dependency of Mcl-1S levels on the cell cycle. **(A,B)** WB analyses of Mcl-1S levels in cells treated with DNA damaging agents at different concentrations for 16 h **(A)**, or at a constant dose of doxorubicin for the different incubation periods **(B)**. A spliceosome inhibitor FR901464 (22 h incubation) was used as a positive control for the identification of the Mcl-1S band on Western blots. Activated p53 (phosphorylated by ATR/ATM at Ser15) served as a marker of the initiation of the DNA damage response **(C)**. RT-qPCR analyses of Mcl-1L and Mcl-1S mRNA levels upon doxorubicin treatment. The data were normalized to control samples and shown as the Mean ± SEM. **(D)** Immunoblot of Mcl-1S protein levels, assessed after 24-h incubation with increasing concentrations of monastrol. **(E)** RT-qPCR analyses of Mcl-1L and Mcl-1S mRNA levels upon monastrol treatment. The data were normalized to control samples and shown as the Mean ± SEM. **(F)** The cell cycle distribution by PI staining following monastrol treatment. The bar heights reflect the mean percentage of cells in G1, S, and G2 phases; all error bars represent the SD. **(G)** Comparison of Mcl-1S mRNA levels in G1/S and G2/M phases of the cell cycle. The cells were synchronized by a double thymidine block and analyzed by flow cytometry and RT-qPCR methods following synchronization (0 h) or after a 6-h release (6 h). **(H)** Mcl-1S protein expression throughout the cell cycle. For each sample, WB and flow cytometry analyses were conducted. The experiments presented in the figure were performed using the HEK293T cell line; the data on the cell cycle generated using other cell lines are presented in Supplementary Figure 2. FR, a spliceosome inhibitor FR901464; M, molecular weight marker; Cntr, vehicle control; SEM, standard error of measurement; PI-A, a propidium iodide fluorescence area. *p < 0.05 compared with corresponding controls.

To examine this hypothesis, we tested several DNA-damaging agents for their ability to raise Mcl-1S levels. Specifically, the HEK293T cells were treated either with different concentrations of cisplatin (3–15 μM), doxorubicin (0.1–2 μM), or etoposide (0.3–8 μM) for 16 h (Figure 1A), or with a constant dose of doxorubicin (0.5 μM) for different incubation periods (Figures 1B,C). Although the initiation of the DNA damage response (as assessed by ATR/ATM-mediated phosphorylation of p53) occurred, no accumulation of Mcl-1S protein with molecular weight (MW) 32 kDa or mRNA was detected under these genotoxic conditions (Figures 1B,C). It should be noted that the level of Mcl-1L mRNA and protein increased after 16 h incubation with 0.5 μM of doxorubicin. According with previously published results, the level of Mcl-1L can be influenced by treatment with low doses of DNA-damaging agents or radiation (Zhan et al., 1997). In contrast to low doses of doxorubicin or etoposide, treatment with 2–8 μM of doxorubicin led to a decrease of the level of 40 kDa Mcl-1L protein but not Mcl-1 mRNA due to the protein degradation via p53-Noxa dependent pathway or caspase-dependent cleavage.

Similarly, another examined stimulus for cell-cycle arrest induction – mitotic spindle disruption by monastrol – was not associated with an increase in the level of Mcl-1S protein. Moreover, at concentrations of 50 μM and higher treatment with monastrol resulted in the decrease of the Mcl-1S protein level after 24 h of incubation (Figure 1D). In striking contrast, monastrol treatment resulted in a pronounced increase in the Mcl-1S mRNA content, as observed both at earlier (4 h) and later (24 h) time points. Noteworthy, cells upon 25 μM monastrol treatment demonstrated a greater upregulation of Mcl-1S mRNA in comparison with that at 100 μM monastrol (Figure 1E). 25 μM monastrol caused only a slight accretion of the G2/M-population compared to the asynchronized control cells, whereas the high dose of this agent (100 μM) effectively arrested cells in G2/M-phase (Figure 1F). These results suggest that accumulation of Mcl-1S mRNA follows both slight perturbations of the cell cycle and the irreversible cell cycle arrest, which could lead to apoptosis. At the same time, the fact that a monastrol-induced splicing switch towards Mcl-1S mRNA was not mirrored at the protein level is rather a consequence of enhanced protein degradation. Indeed, as both Mcl-1L and Mcl-1S are subjected to rapid turnover, their enhanced degradation may abolish the effects of increased mRNA production.

Furthermore, we observed an accumulation of Mcl-1S in the G2/M phases during the normal (or mildly perturbated due to thymidine exposure) cell cycle progression. Mcl-1S mRNA levels, measured at 6 h after the release from a double thymidine block (G2/M phases), were significantly higher compared to the synchronized cells (G1/S phases) (Figure 1G). However, Mcl-1S protein levels only slightly increased during the cell cycle, reaching a peak at the G2/M phases (Figure 1H). The tendency of Mcl-1S mRNA and protein levels to increase upon entry to the G2/M phases also was detected in other non-tumor (fetal lung fibroblasts IMR90) and cancer cell lines (cervical adenocarcinoma HeLa, ovarian carcinoma Caov-4, colorectal carcinoma HCT116, and non-small cell lung carcinoma U1810) (Supplementary Figure 2). Noteworthy, HCT116 and HeLa cells displayed statistically significant increases in Mcl-1S mRNA levels while entering the premitotic stages, whereas other cell lines tended to upregulate Mcl-1S mRNA in a less pronounced manner (Supplementary Figure 2). Additionally, detectable amount of Mcl-1S after block release was found in Caov-4, HCT116 and HeLa cells (Supplementary Figure 2).

In addition, a spindle poison nocodazole (20 nM, 21 h treatment after 2.5 mM thymidine pre-synchronization) was used to induce G2/M-arrest (Supplementary Figure 3). HEK293T cells appeared to be resistant to this concentration of nocodazole up to 8 h after the arrest and have no or a small number of cells in the SubG1-fraction. However, the cells were reluctant to escape nocodazole-induced arrest in the designated time points after the release. Importantly, we also observed the marked decrease in Mcl-1L protein level under these conditions. It could be associated with the p53/Noxa-pathway of Mcl-1L degradation in the DNA-damage conditions (Nakajima et al., 2016). Cells accumulating in 4n fraction at 8 h after the release from the thymidine-nocodazole block were documented, which could suggest that cells gradually developed the state of mitotic catastrophe (Sorokina et al., 2017).

### Mcl-1S Is Capable of Nuclear Translocation

**FIGURE 2:**
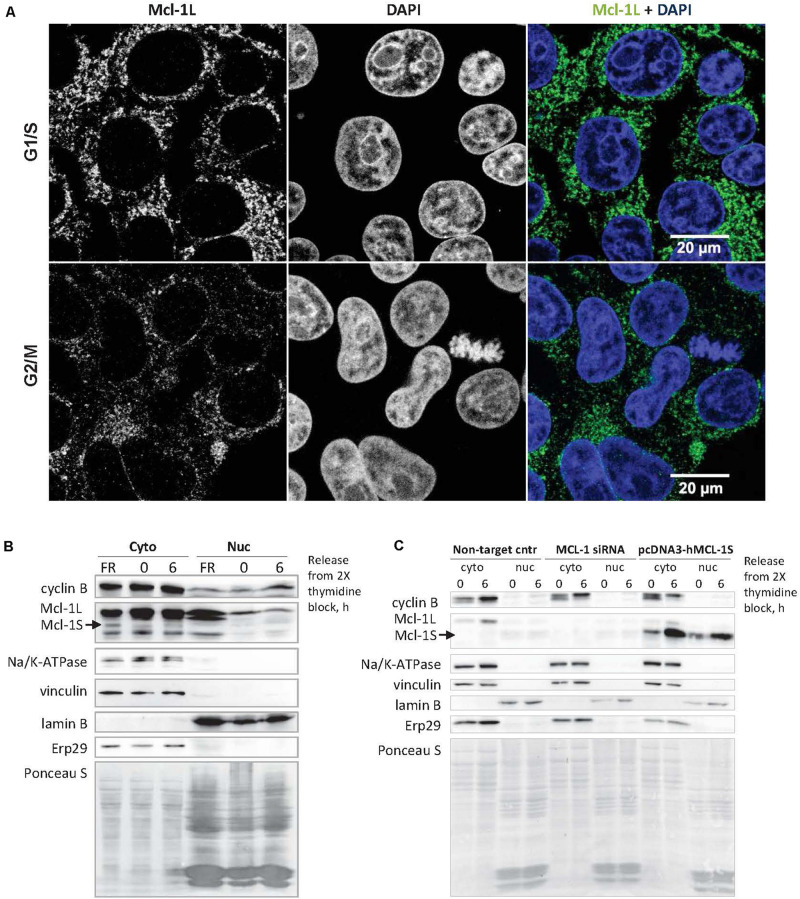
Mcl-1S can translocate into the nucleus, where a low portion of cellular Mcl-1L is also found. **(A)** Confocal fluorescence microscopy of Mcl-1 subcellular localization in G1/S (the upper row of panels) and G2/M phases (the panels below). Nuclear DNA was stained with DAPI (blue). The degree of fluorescence colocalization between Mcl-1 and DAPI in HEK293 cells averaged at 1.04 ± 0.51% (Mean ± SD). Immunoblots of nuclear and cytoplasmic fractionation analysis of the native **(B)** and overexpressed Mcl-1S protein **(C)**. Cyclin B1 was used as a cell cycle progression marker. Na/K-ATPase (a plasma membrane protein), vinculin (a cytoplasmic protein), lamin B (a nuclear protein), ERp29 (an ER protein) served as fractionation markers. Total protein loading was visualized via staining with Ponceau S. Cyto, cytoplasmic extract; nuc, isolated nuclei; Non-target cntr, negative transfection control.

Mcl-1L, the specific endogenous antagonist of Mcl-1S (Bae et al., 2000; Stewart et al., 2010), was shown to translocate into the nucleus to perform the cell cycle-related functions (Jamil et al., 2008, 2010; Thomas et al., 2010). In HEK293T cells, only a low amount (1.04 ± 0.51%, Mcl-1L/DAPI colocalization) of cellular Mcl-1L was detected in the nuclei in the G1/S and G2/M phases according to confocal microscopy (Figure 2A). However, low levels of Mcl-1L may be inhibited even with slightly upregulated amounts of Mcl-1S protein, if Mcl-1S is capable of nuclear translocation. To test the ability of Mcl-1S to internalize into the nuclei, we analyzed the contents after separating the cytoplasmic and nuclear fractions of cells that were synchronized (G1/S phases) and released after a double-thymidine block (G2/M phases). Endogenous Mcl-1S (Figure 2B), as well as Mcl-1S overexpressed with plasmid (Figure 2C) were detected in the nuclear fractions, demonstrating the ability of this protein to enter the nucleus. Moreover, nuclear accumulation was detected after a release from the cell cycle block that might indicate the importance of Mcl-1S in nuclear functions.

### Mcl-1S Overexpression or Mcl-1L Knockdown Causes a Faster Progression Through the S Phase

**FIGURE 3:**
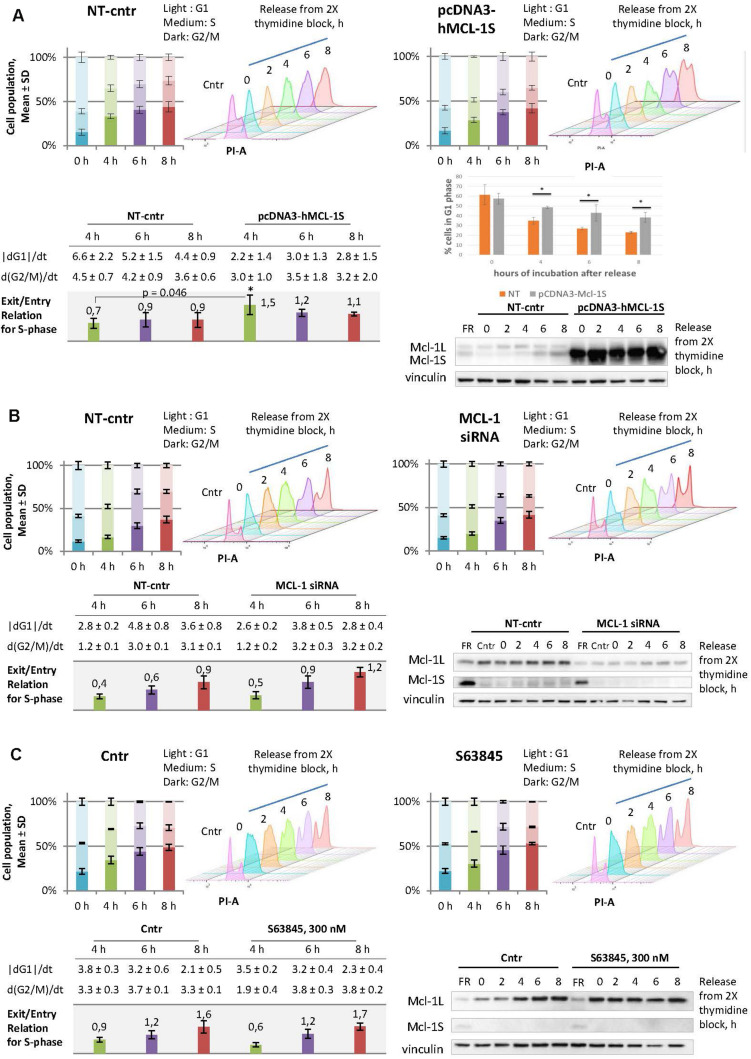
Overexpression of Mcl-1S or siRNA-mediated knockdown of Mcl-1L contribute to the faster progression through S phase, whereas the Mcl-1L chemical inhibitor, S63845, does not. Comparisons between the cell cycle responses to plasmid Mcl-1S overexpression **(A)**, siRNA-mediated knockdown of Mcl-1L **(B)**, or chemical inhibition of Mcl-1L by the BH3-mimetic S63845 **(C)**, and the relative non-target transfections **(A,B)** or vehicle controls **(C)**. HEK293T cells were synchronized by a double thymidine block and analyzed using WB and flow cytometry methods following synchronization (0 h) or after a 2, 4, 6, and 8-h release from the arrest. The time periods of 4, 6, and 8-h release exhibited enough accumulation of a cell population in G2/M phases in comparison to the relative 0-h controls and were chosen for further quantifications. The stacked bar graphs depict the percentage of cells at the G1, S, and G2/M phases; the data are plotted as the Mean ± SD. The tables contain the following calculated (please see the section Materials and Methods: Cell cycle analysis by DNA content) values for each time point (4, 6, and 8-h release): the average rate of exit from G1 phase, the average rate of entry into G2/M phases, and the relation of these two values (exit/entry coefficient for S-phase progression). **(A)** Comparison of non-targeted and Mcl-1S overexpressed cells in G1 phase of the cell cycle, *p < 0.05. Cntr, vehicle control; NT-cntr, non-target negative transfection control; FR, a spliceosome inhibitor FR901464; SD, standard deviation; PI-A, a propidium iodide fluorescence area; dG1/dt, the average rate of exit from G1-phase; d(G2/M)/dt, the average rate of entry G2/M phases. *p < 0.05.

To assess the influence of Mcl-1S on the cell cycle, HEK293T cells were studied at different cell-cycle stages after overexpression of Mcl-1S. The cells were transfected with the plasmid pcDNA3-hMcl-1S for 24 h, then synchronized by a double thymidine block (18 + 9 + 18 h) and analyzed after the different periods of release from the block. The cells abundant in Mcl-1S protein showed a significantly higher rate of the S-phase progression at 4 h and higher rates at 6 or 8 h after release in comparison with the relative control groups (Figure 3A). Meanwhile, overexpression of Mcl-1S led to altered transfer of cells from G1 to S phase in comparison to NT control. Thus, 48% of Mcl-1S overexpressing cells were detected in G1 phase at 4 h after release in comparison to 35% of control cells (Figure 3A). The differences in G1-phase between non-target and Mcl-1S overexpressing cells were statistically significant for all investigated time points (Figure 3A). siRNA-mediated knockdown of Mcl-1L demonstrated a similar tendency: the cells deficient in Mcl-1L progressed into G2/M stages more rapidly, although the increase in the speed of progression was less pronounced (Figure 3B). It should be noted that used siRNA (sense strand: 5′-GCATCGAACCATTAGCAGAdTdT-3′) targeted specifically Mcl-1L transcript (Supplementary Figure 4). The obtained observation could be attributed to insufficient down-regulation of Mcl-1 by siRNA-mediated knockdown. Taking into account the specific antagonistic relationships between Mcl-1L and Mcl-1S (Bae et al., 2000; Stewart et al., 2010), the observed results might suggest that Mcl-1S acts as an inhibitor of Mcl-1L in cell cycle regulation.

In order to examine the potential of a clinical application of an Mcl-1L inhibitor to cause the same cell-cycle perturbations, we tested a BH3-mimetic S63845 under the analogous experimental conditions. In contrast to the genetic treatment, the cells were pre-incubated with S63845 and also incubated with it during synchronization. It is noteworthy that even high concentrations of the compound did not cause apoptosis in the studied cell line (Supplementary Figure 5). It is known that S63845 binding results in the stabilization of Mcl-1L, which is why the accumulation of Mcl-1L serves as an indicator of the inhibitory action of S63845 (Senichkin et al., 2019). Accordingly, 300 nM concentration of S63845 was used in the cell cycle-related experiments, as this dose allowed to obtain the highest accumulation of Mcl-1L (300 nM, Supplementary Figure 5). Somewhat unexpectedly, we did not observe any cell cycle perturbations upon S63845 in our experimental model (Figure 3C). Taken together, the results confirmed the safety of S63845 in the context of cell cycle events at the stated concentration.

### Overexpression of Mcl-1S Leads to DNA Damage Accumulation

**FIGURE 4:**
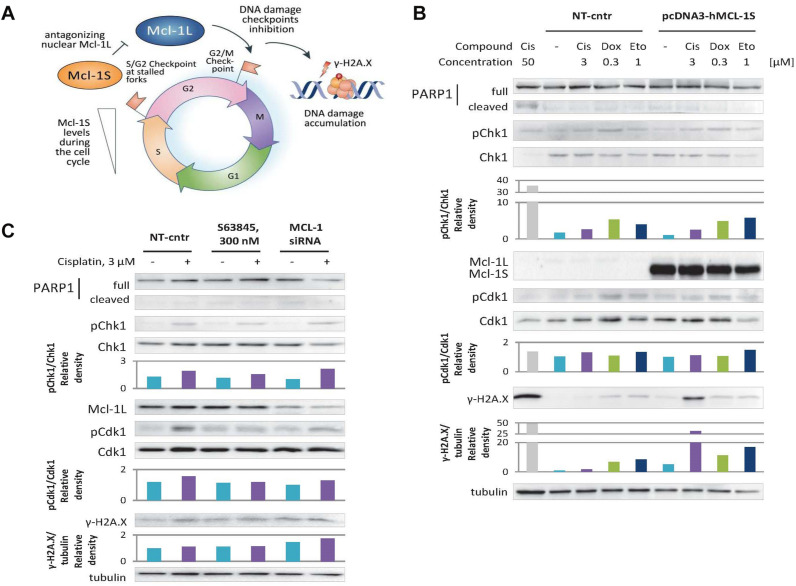
Overexpression of Mcl-1S or siRNA-mediated knockdown of Mcl-1L leads to DNA damage accumulation under normal and genotoxic conditions, while S63845 does not. **(A)** Schematic representation of Mcl-1S function in the cell cycle. WB analyses of γ-H2A.X levels and phosphorylation of the cell cycle regulatory kinases Chk1 and Cdk1 under Mcl-1S plasmid overexpression **(B)** and chemical inhibition or siRNA-mediated downregulation of Mcl-1L **(C)**. Cleavage of PARP1 with the formation of the p89 fragment was used for apoptosis assessment. Cisplatin (50 μM) was used as a positive control for apoptosis. One representative WB analysis out of three independent experiments is shown. Cis, cisplatin; Dox, doxorubicin; Eto, etoposide; NT-cntr, non-target negative transfection control.

We further hypothesized that faster progression through the cell cycle upon Mcl-1S overexpression or Mcl-1L knockdown could be mechanistically explained by decreased activity of kinase Chk1 since Mcl-1L was reported to enhance Chk1 activity by phosphorylation [at the sites: Ser345 (Jamil et al., 2008, 2010; Pawlikowska et al., 2010), Ser317 (Pawlikowska et al., 2010; Mattoo et al., 2017), and Ser296 (Pawlikowska et al., 2010)]. Chk1 is known to mediate S/G2 and G2/M cell cycle arrests by indirectly facilitating Cdk1 phosphorylation at Thr14 and Tyr15 (Patil et al., 2013). Nevertheless, upon Mcl-1S overexpression or Mcl-1L knockdown, we did not observe any significant reduction in the activity of Chk1 (as assessed by Chk1 phosphorylation at Ser345) or Cdk1 (based on phosphorylation of Tyr15) (Figures 4B,C). Taken together, Mcl-1S-mediated cell cycle regulation is not dependent on Chk1 activity in this experimental model.

Nevertheless, the revealed faster progression into mitosis upon Mcl-1S overexpression or Mcl-1L knockdown might occur due to the disruption of premitotic DNA damage checkpoints (S/G2 or G2/M checkpoints). If so, more DNA damage accumulation should be observed in these cells as compared to that of the control groups (Figure 4A).

To test whether the cells abundant in Mcl-1S accrue more DNA damage than control cells, we performed 16-h incubations in non-genotoxic conditions, and also with such concentrations of DNA damaging agents that are sufficient to induce a DNA damage response (as assessed by phosphorylation of p53 at Ser15 site), but do not influence Mcl-1L protein levels (3 μM cisplatin, 0.3 μM doxorubicin, or 1 μM etoposide) (Figure 1A). Indeed, the cells transfected with pcDNA3-hMcl-1S plasmid demonstrated a higher degree of H2A.X phosphorylation at Ser139 (γ-H2A.X), considered as a marker of DNA damage, than those transfected with an empty vector, both in non-cytotoxic conditions and upon genotoxic stress. The treatment with doxorubicin and etoposide resulted in 2–3-fold higher γ-H2A.X accumulation upon Mcl-1S overexpression in contrast to control cells. Interestingly, a 25-fold increase in H2A.X phosphorylation levels was detected upon cisplatin treatment, which confirmed the extremely pronounced accumulation of DNA damage in the cells overexpressing Mcl-1S (Figure 4B). The DNA damage accumulation was higher in cells with siRNA-mediated knockdown of Mcl-1L than in the non-target group. However, the increase in DNA damage marker was less pronounced compared to Mcl-1S overexpression, which could be attributed to the insufficient efficiency of the gene-silencing treatment (Figure 4C). Noteworthy, a BH3-mimetic S63845, which was shown not to influence the cell cycle progression in the above experiments, did not affect DNA damage accumulation as well. Overall, the results demonstrate that upregulation of Mcl-1S results in DNA damage accumulation, whereas inhibition of Mcl-1 promotes either a slight increase in DNA damage (siRNA knockdown) or no change at all (chemical inhibition with 300 nM S63845).

## Discussion

Mcl-1S was shown to be a selective endogenous inhibitor of Mcl-1L in apoptosis (Bae et al., 2000; Stewart et al., 2010). However, it remains unclear what apoptotic stimuli trigger Mcl-1S upregulation. Moreover, while many other pro-apoptotic proteins are known to antagonize survival functions of Mcl-1 (Senichkin et al., 2019), the pro-death role of Mcl-1S seems to be redundant in the cell. Arguably, the cell could also exploit Mcl-1S for other processes, and the highly selective interaction between Mcl-1S and Mcl-1L might lie beyond apoptotic regulation.

In this work, we first focused on the proapoptotic trigger that could lead to an increase in Mcl-1S levels. Based on previously published data (Moore et al., 2010), we assumed that strong activation of G2/M or the spindle-assembly checkpoint would result in proapoptotic splicing of Mcl-1. Indeed, mitotic spindle disruption by monastrol was associated with a moderate increase in Mcl-1S mRNA production. However, surprisingly, this effect was not translated into changes in protein level, while high concentrations of monastrol resulted in a drop of Mcl-1S level. Moreover, the more pronounced increase in Mcl-1S mRNA was observed at the concentration of monastrol that failed to provoke an effective G2/M arrest, but only slightly augmented the percentage of cells in G2/M phase (Figures 1D–F). This augmentation was maintained after more prolonged incubation (24 versus 4 h) with monastrol, suggesting the reversibility of the G2/M arrest in these conditions and the lack of proapoptotic conditions (Figure 1F).

Further analysis of the cell cycle course after thymidine synchronization revealed a similar result of mRNA Mcl-1S accumulation in cells reaching premitotic stages (Figures 1G,H and Supplementary Figure 2). Notably, Mcl-1L also demonstrated cell cycle-dependent increases in protein concentration, whereas its mRNA levels oscillated differently in the examined cell lines. Thus, it seems that Mcl-1L upregulation during the cell cycle generally takes place at the post-translational level. Therefore, the appearance of Mcl-1S mRNA is not “an artifact” of the increased transcription of the *MCL1* gene as could be presumed by the kinetic model of co-transcriptional AS (Kim et al., 2017). Overall, the above results suggest a specific non-apoptotic role of Mcl-1S during cell cycle progression.

Given the fact that Mcl-1S is a selective intracellular antagonist of Mcl-1L (Bae et al., 2000; Stewart et al., 2010) and that Mcl-1L participates in cell cycle regulation (Senichkin et al., 2019), it was logical to assume the negative control of Mcl-1L by Mcl-1S during cell cycle progression. However, the cellular levels of Mcl-1L protein were substantially higher than those of Mcl-1S as determined by Western blot assays of whole-cell lysates. Compartmentalization could serve as an explanation for the possibility that small amounts of Mcl-1S inhibit the nuclear function of Mcl-1L during cell cycle progression. The levels of Mcl-1L in the nucleus, where it interacts with the cell cycle regulators, are low enough (Figure 2A) and thus could be efficiently antagonized by Mcl-1S accumulation. Moreover, we demonstrated that native and overexpressed Mcl-1S could localize in the nuclear compartment, including during the G2/M stages, thus highlighting the possibility of Mcl-1L and Mcl-1S antagonism during cell cycle control (Figures 1B,C).

Our additional experiments revealed that overexpression of Mcl-1S and siRNA-mediated knockdown of Mcl-1L had the same tendency in cell cycle responses (Figures 3A,B). The cells abundant in Mcl-1S or deficient in Mcl-1L showed accumulation in G1 phase (Figure 3A), while they maintained a similar level of the G2/M population (in comparison to relative non-target controls). This statement can be explained by the G1 arrest of overexpressing Mcl-1S cells and their faster progression of cell cycle from G2/M to G1 phase. This could only occur due to a faster progression through S phase of the cells which had entered this stage. Importantly, a low-molecular weight inhibitor of Mcl-1L, S63845, did not cause the same response (Figure 1C). There are at least two possible explanations of this result. First, Mcl-1L could perform its cell cycle role independently of the BH3-binding groove to which S63845 interacts. This notion further implies the interaction between Mcl-1S and Mcl-1L to be more or less different from the canonical BH3-domain binding to the hydrophobic groove. However, there is no structural data of the interaction between full-size Mcl-1L and Mcl-1S available to confirm or disprove this assumption. Second, S63845 could fail to efficiently antagonize the nuclear fraction of Mcl-1L in HEK293T cells due to the abundance of cytoplasmic Mcl-1L molecules, which could hinder S63845 subcellular distribution into the nucleus (Kotschy et al., 2016). Overall, these results demonstrate that S63845 does not influence the cell cycle progression and thus cannot cause the related side effects (such as DNA damage accumulation in non-target cells), whereas the upregulation of Mcl-1S may provide the cell cycle perturbations apparently via the antagonism with Mcl-1L. Additionally, it is not unlikely that Mcl-1S possesses Mcl-1L-independent functions during cell cycle regulation.

We assumed that the Chk1/Cdk1 axis is the major pathway by which Mcl-1 isoforms affect the cell cycle because Mcl-1L was reported to modulate the activity of Chk1 (Jamil et al., 2008, 2010; Pawlikowska et al., 2010; Mattoo et al., 2017). However, we observed only an insignificant decrease in Cdk1 phosphorylation (Tyr15) and no tendentious changes in Chk1 phosphorylation (Ser345) upon Mcl-1S overexpression or Mcl-1L knockdown (Figures 4B,C). It, thus, appears that the influence of Mcl-1 isoforms on the cell cycle is rather independent of Chk1 activity in our experimental model.

Nevertheless, the faster progression through premitotic stages implies the abolishment of S/G2 (at stalled DNA replication forks) and/or G2/M checkpoints for DNA damage repair. Consequently, cells abundant in Mcl-1S or deficient in Mcl-1L may accumulate more DNA lesions than control cells (Figure 4A). Indeed, we showed that overexpression of Mcl-1S or siRNA-mediated knockdown of Mcl-1L led to higher levels of the DNA damage marker γ-H2A.X both under control conditions and genotoxic treatment. Consistently, treatment with S63845 did not result in the same changes (Figures 4B,C). This provides evidence for the safety of S63845-like compounds in the context of cell cycle-related adverse effects, which serves as a good sign of their potential therapeutic applications.

## Data Availability Statement

The raw data supporting the conclusions of this article will be made available by the authors, without undue reservation, to any qualified researcher.

## Author Contributions

AS performed experiments (presented in Figures 1, 2A, 4B,C and Supplementary Figures) and wrote the text (sections “Materials and Methods” and “Results”). VS performed experiments (presented in Figures 2B,C, 3) and wrote the text (abstract, sections “Introduction” and “Discussion”). TP and TZ performed synthesis of all siRNAs. BZ responsible for the initial concept of the study, study supervision and checking the text. GK responsible for experimental design, study supervision and checking the text. All authors contributed to the article and approved the submitted version.

## Conflict of Interest

The authors declare that the research was conducted in the absence of any commercial or financial relationships that could be construed as a potential conflict of interest.
