# Supplementary material for: Upregulation of Mcl-1S Causes Cell-Cycle Perturbations and DNA Damage Accumulation
Source: Front Cell Dev Biol. 2020 Sep 25;8:543066. doi: 10.3389/fcell.2020.543066 (PMC7544834; doi:10.3389/fcell.2020.543066)
Supplement: Supplementary file 1 [file Data_Sheet_1.PDF]

Supplementary Material

1. Supplementary Figures

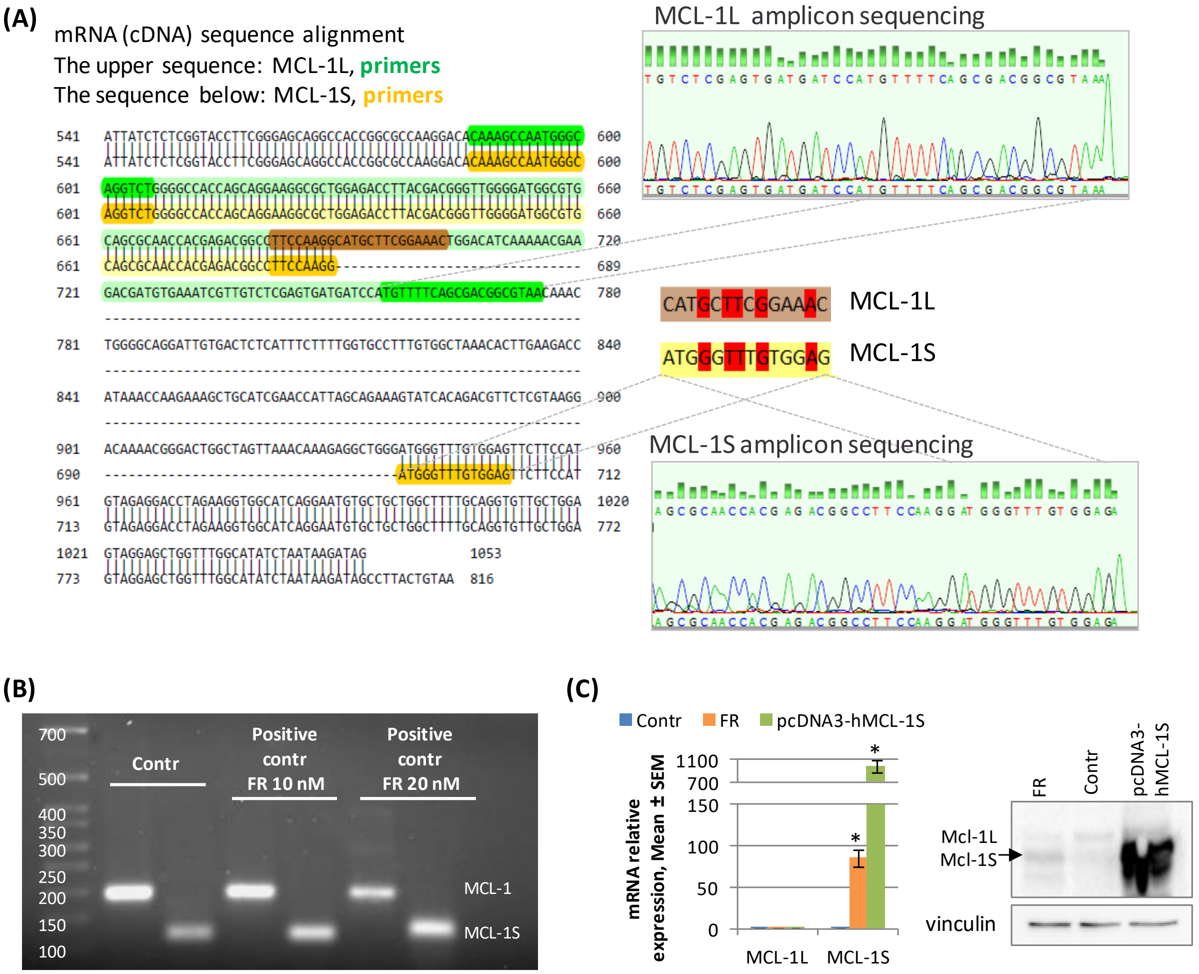

Supplementary Figure 1. Experimental settings for the detection of Mcl-1L and Mcl-1S isoforms. **A.** Sequencing of the Mcl-1S and Mcl-1L amplicons to confirm qPCR primer specificity. On the left: the sequence alignment of Mcl-1L and Mcl-1S mRNAs (5' → 3', starting with 541 base pair). The template regions for Mcl-1L primer annealing are highlighted in bright green, while the remaining amplicon sequence is presented in light green; the same data for Mcl-1S are depicted in yellow; the splice-junction of exons 1 and 2 is marked in brown. On the right, top to bottom, the sequence data of the Mcl-1L fragment, the exon splice junction areas for both transcripts with nucleotide similarities are highlighted in red, the sequence data for Mcl-1S amplicon as visualized by

DNA Baser Sequence Assembler (Heracle Biosoft Srl). **B.** Separation of amplification products by 2% agarose gel electrophoresis to confirm qPCR primer specificity. The length of the amplicons: Mcl-1L (189 bp) and Mcl-1S (117 bp). A spliceosome inhibitor FR901464 was used for the switching of Mcl-1 pre-mRNA AS towards the Mcl-1S isoform. **C.** Mcl-1S and Mcl-1L mRNA and protein levels, as assessed by RT-qPCR (on the left) and WB analysis (on the right), upon FR901464 treatment or transfection of the plasmid pcDNA3-hMcl-1S. $^{*}p < 0.05$

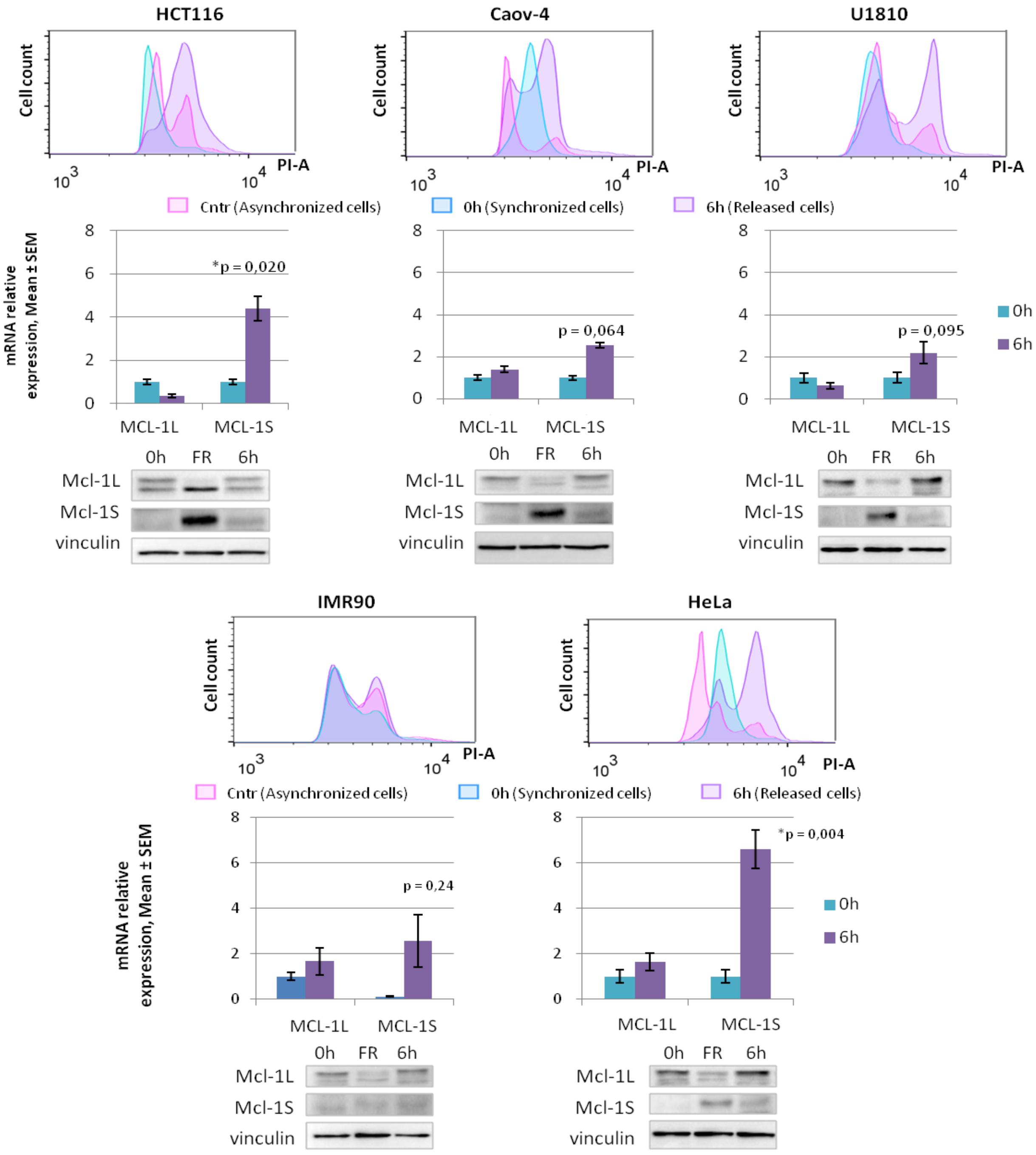

Supplementary Figure 2. Mcl-1S mRNA and protein levels tend to increase in G2/M phases in different cell lines. The following cell lines were analyzed: the human fetal lung fibroblasts IMR90, cervical adenocarcinoma HeLa, ovarian carcinoma Caov-4, colorectal carcinoma HCT116, and non-small cell lung carcinoma U1810. The cells were examined by flow cytometry with PI staining, RT-qPCR and WB techniques upon cell-cycle synchronization by a double thymidine block (0 h) or 6-

hour release from the arrest. RT-qPCR data were normalized to control samples (0 h) and are shown as the Mean ± SEM. * $p < 0.05$

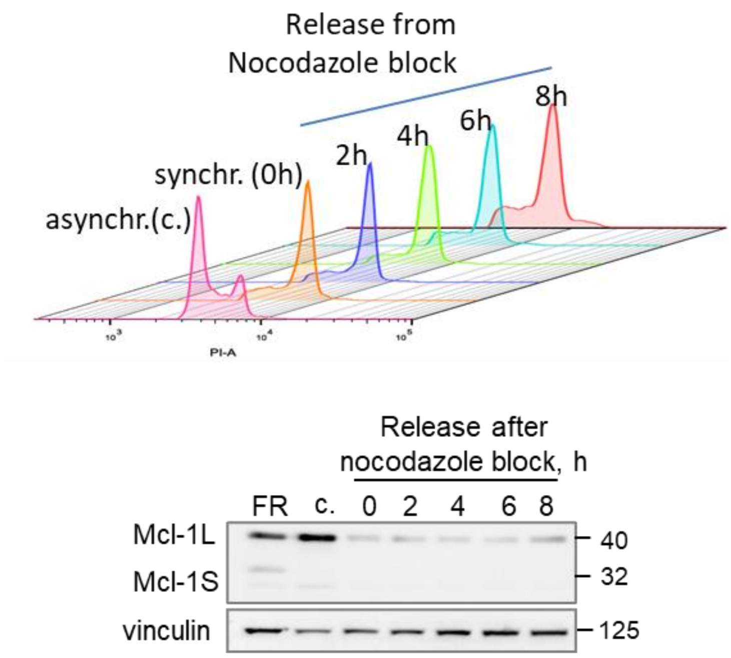

Supplementary Figure 3. HEK293T were reluctant to escape nocodazole-induced arrest. HEK293T cells were synchronized by 20 nM of nocodazole during 21 h treatment after 2.5 mM thymidine pre-synchronization and analyzed using WB and flow cytometry methods following synchronization (0 h) or after a 2, 4, 6 and 8-h release from the arrest.

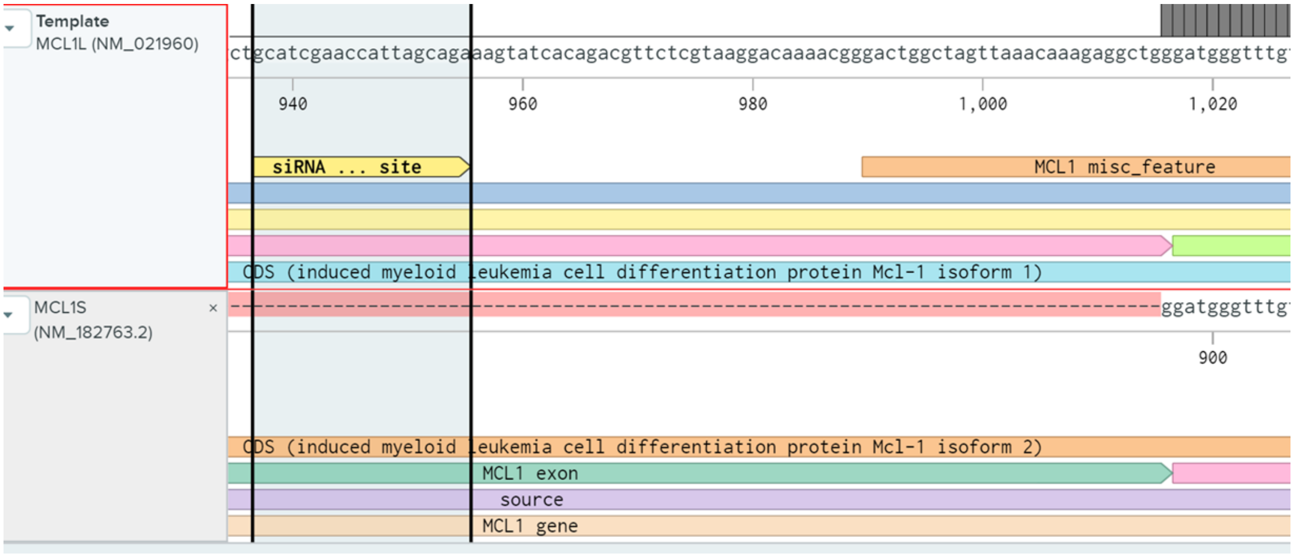

Supplementary Figure 4. siRNA (sense strand: 5′-GCATCGAACCATTAGCAGAdTdT-3′) against Mcl1 transcripts targets specifically Mcl1L transcript. Alignment of Mcl1L (the upper panel) and Mcl1S (the bottom panel) mRNA-transcripts (NCBI Genbank accession codes NM_021960.5 and NM_182763.2, respectively) was performed in Benchling software (Benchling, Inc., San Francisco, USA). Misalignments are shown in red. The dashes represent the absence of nucleotides in MCL1S sequence; deletions correspond to the absence of exon 2 in Mcl1S. siRNA-target site (‘siRNA… site’, 5′-GCATCGAACCATTAGCAGA-3′) is annotated in yellow and highlighted by selection (black lines). Other annotations are generated by NCBI Genbank.

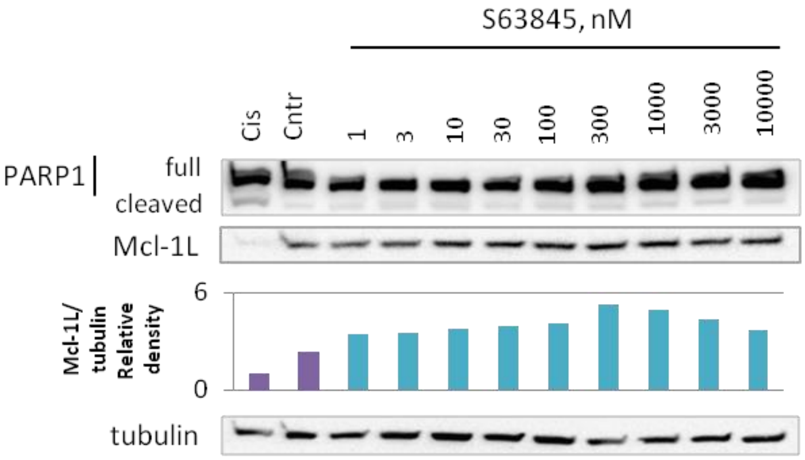

Supplementary Figure 5. Increasing concentrations of S63845 do not induce a change in the level of an apoptotic marker (cleaved PARP) in HEK293 cells. The cells were incubated with the stated concentrations of S63845 for 24 h. Cisplatin (50 µM) was used as a positive control for apoptosis; Cntr represents a vehicle control (DMSO).
